# Contraceptive counseling for adolescents in the emergency department: A novel curriculum for nurse practitioners and physician assistants

**DOI:** 10.1097/JXX.0000000000000824

**Published:** 2023-09-01

**Authors:** Laurel S. Gabler, Michelle Shankar, Tara Ketterer, Jennifer Molnar, Amber Adams, Jungwon Min, Elizabeth Miller, Romina L. Barral, Aletha Akers, Melissa K. Miller, Cynthia Mollen

**Affiliations:** 1 Pediatric Emergency Medicine, Boston Children’s Hospital, Boston, Massachusetts; 2 Department of Pediatrics, Children’s Hospital of Philadelphia (CHOP), Philadelphia, Pennsylvania; 3 Policylab, CHOP, Philadelphia, Pennsylvania; 4 Emergency Medicine, CHOP, Philadelphia, Pennsylvania; 5 Churches United for Justice, Saint Louis, Missouri A. Adams was a research associate at Children’s Mercy Hospital, Kansas City, Missouri; 6 Department of Biomedical and Health Informatics, CHOP, Philadelphia, Pennsylvania; 7 Division of Adolescent and Young Adult Medicine, UPMC Children’s Hospital of Pittsburgh; Professor of Pediatrics, Public Health and Clinical and Translational Science, University of Pittsburgh School of Medicine, Pittsburg, Pennsylvania; 8 Division of Adolescent Medicine, Children’s Mercy Hospital and Clinics, Kansas City, Missouri; Assistant Professor of Pediatrics, University of Missouri-Kansas City School of Medicine, Kansas City, Missouri; Research Assistant Professor of Pediatrics, University of Kansas School of Medicine, Kansas City, Missouri; 9 The Guttmacher Institute, Philadelphia, Pennsylvania; 10 University of Missouri-Kansas City; Attending Physician, Children’s Mercy Hospital, Kansas City, Missouri; 11 Attending Physician, Emergency Medicine, CHOP, Philadelphia, Pennsylvania; Professor of Pediatrics, Perelman School of Medicine at the University of Pennsylvania; Distinguished Chair, Department of Pediatrics, Philadelphia, Pennsylvania

**Keywords:** adolescent health, contraception, contraceptive counseling, emergency department, teen pregnancy

## Abstract

Many adolescents use the emergency department (ED) as their primary source of health care. As a result, the ED serves as a unique opportunity to reach adolescents. Although many adolescent visits to the ED are related to reproductive health, ED providers report barriers to providing this care, including lack of training. Nurse practitioners (NPs) and physician assistants (PAs) serve a vital role in the provision of consistent care to adolescents in the ED. The purpose of this study was to create a curriculum to train NPs and PAs at two pediatric institutions to provide patient-centered contraceptive counseling to adolescents in the pediatric ED regardless of their chief complaint. To do this, we created a four-part webinar followed by an in-person training session. Participants completed training and then conducted counseling sessions with adolescents in the ED. Counseling sessions were recorded and reviewed for fidelity to delineated counseling principles, and data from post-counseling surveys were collected. 27 NPs and PAs completed the training and conducted 99 counseling sessions. Nearly all sessions incorporated essential content and communication principles such as shared decision making (90%) and teach-back methods (75%). All NPs and PAs who participated reported satisfaction and subjective improvement in knowledge and competence from the training. This curriculum offers a novel and feasible approach to train NPs and PAs to deliver patient-centered contraception counseling to adolescents in the ED setting, and it can serve as a model for how to educate different providers to incorporate reproductive health education into the busy ED visit.

## Introduction

For many adolescents, the emergency department (ED) is the primary place where they receive medical care (Lehmann et al., 1994; Ziv et al., 1998). This is particularly true for vulnerable adolescents who face multiple barriers in accessing primary care, such as transportation, cost, and confidentiality concerns (Klein et al., 1998; Miller et al., 2013). Those using the ED as their main source of care have been found to be more likely to engage in risky sexual behaviors, have unprotected sex, have multiple sexual partners, and abuse substances (Miller et al., 2013; Wilson & Klein, 2000). As such, the ED is an excellent environment to reach vulnerable adolescents and offer counseling that they might not otherwise receive. If they do not receive this education in the ED, they may not receive it anywhere else. For adolescents who do visit their primary care providers, less than 36 seconds of each health visit is actually dedicated to reproductive health education, and fewer than half of reproductive-aged women receive family planning services in the primary care setting (Akers et al., 2010; Alexander et al., 2014). Prior studies have demonstrated the success of offering counseling for injury prevention, mental health, or alcohol abuse to teens presenting to the ED (Grupp-Phelan et al., 2012; Johnston et al., 2002; Spirito et al., 2004). Regarding reproductive health specifically, several studies involving adolescents seeking care in the ED have demonstrated the desire for and acceptability of offering contraceptive counseling and services in this environment (Caldwell et al., 2020; Chernick et al., 2015; Gutman et al., 2020; Hoehn et al., 2019). One single-site study demonstrated initiation of contraception in 25% of adolescent female participants who received counseling in the ED (Hoehn et al., 2019).

Despite significant potential for the pediatric ED to serve as an important setting for adolescents to access essential contraceptive care, there is limited literature regarding this topic. Although there are several curricula for training providers or trainees on topics relating to contraception, there are no curricula or trainings aimed specifically to teach nurse practitioners (NPs) or physician assistants (PAs) to deliver contraceptive counseling to adolescents in the busy pediatric ED setting (Maciuba & Chen, 2019; Worthington et al., 2020). Therefore, the goal of this study was to develop and evaluate a novel curriculum consisting of webinar modules and an in-person session to train NPs and PAs to provide contraceptive counseling to adolescents in the pediatric ED. Not only is this curriculum unique, but it also fills a much needed void by empowering providers to ultimately empower vulnerable teenagers whose reproductive health may otherwise be completely overlooked by the existing health care system.

## Methods

### Study design

We convened a group of pediatric emergency medicine physicians and adolescent health experts from the two participating institutions to develop learning objectives of the training (Table 1). We designed a four-part, self-paced webinar to provide background information, followed by an in-person training session to allow for content clarification and hands-on practice with observed role plays. Participants were also provided a resource packet for use during the counseling sessions and were sent weekly emails that provided contraceptive facts.

We drew from existing training materials in the published and grey literature to create a training program that would deliver informative, developmentally appropriate, and succinct contraceptive counseling to adolescents within the ED setting (“Committee Opinion No. 710,” 2017; Gavin et al., 2014; Jaccard & Levitz, 2013; Maciuba & Chen, 2019; Ott et al., 2014; Rinehart et al., 1998). Background information on teen pregnancy was obtained from the Guttermacher Institute (https://www.guttmacher.org/fact-sheet/american-teens-sexual-and-reproductive-health). General content for the training program was derived from resources obtained from the Reproductive Health National Training Center (https://rhntc.org/), the Contraceptive Action Plan (https://contraceptiveactionplan.org/), the University of California, San Francisco’s “Beyond the Pill” program (https://beyondthepill.ucsf.edu/), and The Adolescent Health Working Group’s *Sexual and Reproductive Health Toolkit for Adolescent Providers* (Monasterio et al., 2010). Specific information on contraceptive options was obtained from Bedsider.org (https://www.bedsider.org/birth-control), the Reproductive Health Access Project (https://www.reproductiveaccess.org/contraception/), Washington University School of Medicine’s Contraceptive Choice Center (https://contraceptivechoice.wustl.edu/birthcontrol-methods/), and Hatcher’s (2018) *Contraceptive Technology*. In addition to general contraceptive information, the trainings included content regarding discussion of confidentiality, developmentally appropriate communication strategies such as open-ended questioning and teach-back methods, and patient-centered counseling techniques that incorporated principles of shared decision making and cultural competency.

#### Training webinars.

The webinars were divided into four modules: (1) background information on teen pregnancy including general statistics on teen pregnancy and contraception usage, (2) theoretical frameworks underlying contraception choice, (3) contraception options, and (4) patient-centered counseling techniques. They were designed to be completed in about 2 hours (see Table 2, for an overview of the webinar modules). Adolescent health experts across the two institutions reviewed the webinar content for accuracy and suitability. Participants received an email containing a link to the online webinars one month in advance of the in-person training session and were asked to complete the modules before the in-person session.

#### In-person training session.

This four-hour session was designed to reinforce and clarify content of the webinars and allow for hands-on counseling practice. It was led by study investigators with pediatric emergency medicine and adolescent health expertise. The session began with a presentation to review webinar content. Next, participants observed and discussed counseling, including a demonstration led by an adolescent medicine health expert. Finally, participants engaged in role-play scenarios created by a study team member with pediatric gynecology and adolescent health expertise that were adapted for application in the ED setting. Participants played the roles of observer, provider, and patient one time each (Table 3). Participants were divided into groups of three and asked to switch roles during each scenario. The session concluded with time to answer outstanding questions and review procedures for post-counseling session follow-up.

After the in-person sessions, all participants received an email containing links to the presentation and presentation resources, the webinar, a map of local family planning clinics, a list of Title X clinics, and a guide with additional contraception resources. All participants then went on to conduct actual counseling sessions with adolescents in the ED.

### Study setting and population

This curriculum was developed as part of a National Institutes of Health–funded study to assess intention to initiate contraception among adolescents who receive ED-based contraceptive counseling. It was performed in two EDs affiliated with large academic pediatric medical centers between January 2019 and February 2021. Collectively, these pediatric EDs care for more than 170,000 patient visits annually. Both sites are staffed with pediatric emergency medicine trained attendings and fellows; residents; and NPs and PAs, who care for lower acuity (triage ESI level 4 and 5) patients primarily and staff more severely ill and injured patients with the attending. Before this study, there was no formal contraceptive counseling training for ED providers at these institutions.

We chose ED NPs and PAs as our target learners because they are experienced clinicians who provide consistent frontline care in the ED setting and have some flexibility in their care provision because they do not have responsibility for the overall ED management. The NPs and PAs at these institutions also see the bulk of the lower acuity adolescent patients, which makes them the ideal providers to receive this training. NPs and PAs with a minimum of 6 months of experience in the pediatric ED at the two study institutions were eligible to participate in the trainings. No prior contraceptive counseling experience was necessary. Participants were recruited through email and in person through divisional meetings.

### Target audience for counseling

Adolescents seeking care in one of the two participating pediatric EDs were eligible for counseling sessions if they were (1) female participants aged 15–18 years; (2) high risk for unintended pregnancy (defined as having had consensual sexual activity within the past 6 months) or expressing likely future sexual activity (defined as sexual activity within the following 3 months); (3) fluent in English; (4) not planning to get pregnant in next 12 months; and (5) not currently using hormonal contraception or long-acting reversible contraception.

Adolescents were not eligible for counseling if they had (1) presented to the ED after a sexual assault, (2) tested positive for pregnancy, (3) appeared too ill to be screened, (4) exhibited a developmental delay that could affect participation, or (4) had been deemed by the treating provider as inappropriate for enrollment. Consent or assent for counseling was obtained as approved by the institutional review board (IRB). All counseling sessions took place in private and were audio recorded by the provider.

### Study protocol

All NP and PA participants provided written consent to participate, and the study was approved by the lead site IRB.

Evaluation of the training consisted of an adolescent medicine expert at each site providing an objective review of randomly selected audio recordings using a standardized fidelity form and of provider self-report. Table 4 provides an overview of the educational objectives of the trainings and the evaluation process.

### Key outcome measures

To assess the success of the curriculum, we used a combination of third-party observation of the counseling sessions and anonymous participant self-completed surveys to evaluate whether providers were able to meet the curricular learning objectives in their counseling sessions with adolescents. In addition, we used anonymous participant surveys to gauge participants’ overall impressions of the trainings at the end of each counseling session and at the end of the study period.

Approximately half of the audio recordings at each site were randomly selected to be reviewed for adherence to training content and principles using a standardized form. Fidelity assessment included the participant’s ability to establish rapport with the patient and demonstrate empathy; explain confidentiality principles and contraception side effects and risks; use a tiered-effectiveness approach; demonstrate the teach-back method; use open-ended questions; consider the patient’s cultural background; use shared decision-making principles; create a safe environment; incorporate the patient’s personal goals; and present medically accurate information.

After each counseling session, the provider (NP or PA) completed a 17-item feasibility survey containing Likert scale and yes/no response items to self-report ease, satisfaction, competence, and confidence with conducting the counseling sessions. At the conclusion of the study, participants also completed an optional post-study survey, including Likert scale and free response questions, regarding overall impressions of the training and counseling sessions.

### Data analysis

Descriptive statistics were run on all quantitative survey data using Stata version 16 (Statacorp, College Station, TX). All free-response survey answers were manually coded to extract emerging themes. Coding was used to categorize and summarize the main themes that emerged from these responses.

## Results

A total of 27 NPs and PAs completed the training, including 16 providers at site 1 and 11 providers at site 2. Most participants (91%) had minimal to no prior contraceptive counseling experience. Of the providers who were trained, 14 providers at site 1 and nine providers at site 2 conducted one or more sessions during the study period. The mean number of counseling sessions per individual provider was four at site 1 (range 0–6) and five at site 2 (range 0–10). Overall, 99 total individual counseling sessions were completed.

Participants completed postcounseling feasibility surveys for 96 of the 99 sessions. Audio recordings of 50 sessions were reviewed by an adolescent health expert (study team members) for fidelity to the counseling principles. Twelve of the participating providers (44%) completed the post-study survey.

### Evaluation of success in meeting stated learning objectives

Learning Objective 1: Discuss the risks, benefits, and side effect profiles of the different contraceptive options with adolescents using a tiered-effectiveness approach

Nearly all reviewed counseling sessions included an overview of the risks, benefits, and side effects profiles of the various contraceptive methods (98%, n = 49). Furthermore, most sessions were noted to include a tiered-effectiveness approach to prioritize the most effective methods (92%, n = 46).

Learning Objective 2: Succinctly counsel adolescents in the ED regarding various contraceptive options

Mean duration of counseling sessions was 12 minutes. In addition, 87% (n = 84) of providers reported having adequate time during the ED visit to include the necessary information in their session.

Learning Objective 3: Ask open-ended questions and demonstrate the principles of teach-back in providing counseling

In the reviewed sessions, nearly all participants asked open-ended questions (96%, n = 48) and used teach-back methods (76%, n = 38) in their counseling.

According to one NP, “I used [teach-back] at the end of my counseling sessions to tie together a plan with the teen so they could tell me what they liked and their plans for following up.”

Learning Objective 4: Use patient-centered techniques, cultural competency and shared decision-making principles to create a safe environment in which to guide adolescents in making contraceptive choices

In most of the reviewed counseling sessions, providers were noted to have created a safe environment through discussion of confidentiality (96%, n = 48), discussed personal goals of the adolescent (96%, n = 48), and used shared decision making to establish a contraceptive plan (92%, n = 46).

According to one NP, “In each [counseling] session after going through contraceptive choices we would go through each method and the participant would tell me what she thought about it—what she liked, didn’t like or why it may or may not work for her at that time.”

Only 12% (n = 6) of reviewed sessions, however, included an explicit discussion of cultural background in making decisions regarding contraception. In assessment of the training sessions in the final survey, one NP commented, “I don’t remember receiving cultural specific trainings.”

### Counselor self-reported satisfaction

Participants reported feeling satisfied (85%; n = 84) and competent (86%, n = 85) after each counseling session. Of those who conducted more than one session, the majority felt their confidence improved as they gained more experience with the counseling (90%, n = 83).

### Overall training feedback

In the poststudy survey, all the respondents (100%) reported being satisfied with the training. They felt that the training contributed to their professional growth and advancement. All respondents also felt that the trainings increased their overall knowledge regarding contraception and sexual health. All of the respondents felt competent and knowledgeable in their understanding of contraceptive choices after participation.

According to one of the NPs, “[The trainings] helped increase my knowledge of contraceptive choices. I never offered them to patients prior. Now I talk about it with lots of patients and have multiple patients start Depo and seek follow-up care when that wasn’t even their chief complaint!”

According to another NP, “[From the trainings] I learned a lot about the different types [of contraception], and my general sex education counseling for patients [in the ED] improved dramatically.”

In addition to improved knowledge regarding contraception, several of the providers (33%) felt that the training sessions helped them to communicate more effectively with adolescents, thus enabling them to be better partners in the shared decision-making process:

“Being able to learn [from the trainings] how to communicate more effectively with adolescents allowed me to partner with patients better.”

According to another NP, “[The trainings] taught me words and phrases to use with the patients that helped build trusting relationships.”

Several participants (33%) felt the trainings lacked content regarding side effects and contraindications to various contraceptive methods, and others (25%) would have liked more training specifically on intrauterine device and contraception implant insertions. Other recommendations included additional tips to make the counseling more engaging and accessible for teens as well as time management skills and strategies to balance counseling with the competing demands of the busy ED environment.

With respect to the duration of webinar modules, half of the participants (50%) liked that the webinar could be viewed on their own time, whereas others (42%) felt the recorded webinar videos were too slow and would have benefitted from being shorter in duration.

All participants (100%) appreciated the in-person session. Many found the session to be engaging and interactive, appreciated the mock counseling practice, and valued learning from an adolescent provider.

## Discussion

Although several contraceptive counseling curricula for medical providers exist (Maciuba & Chen, 2019; Worthington et al., 2020), this training is novel in that it is both specifically tailored to the ED setting by emphasizing strategies to counsel succinctly while still ensuring patient centeredness and shared decision making, as well as that it is specifically tailored to training NPs and PAs, who provide most care for adolescent patients at many medical centers. The ED has already proven to be an ideal setting in which to counsel adolescent patients to successfully bring about behavior change (Grupp-Phelan et al., 2012; Johnston et al., 2002; Spirito et al., 2004), and prior studies have demonstrated the acceptability and desire for contraceptive counseling in this setting (Caldwell et al., 2020; Chernick et al., 2015; Hoehn et al., 2019). This study adds to that body of work and provides a roadmap for how to efficiently train ED frontline providers to conduct contraceptive counseling with vulnerable patients and also offers a rubric for how to evaluate the success of those trainings. Our work provides a foundation on which additional ED-specific trainings for NPs and PAs can be built. Furthermore, ED-based NPs and PAs can serve as an important bridge to contraceptive providers in the community. This study underscores the critical role that ED-based NPs and PAs play in patient education and counseling in this setting and emphasizes the importance of and flexibility of their roles beyond the direct provision of medical care.

Although NPs and PAs have a critical role to play in this counseling, this training has broader applicability to other clinicians and environments beyond the NPs, PAs, and the two centers where it was initially implemented. More, there is a great need for this type of training given that few pediatric providers actually possess this expertise or are comfortable discussing family planning with patients (Akers et al., 2010; Dehlendorf et al., 2010; Fuzzell et al., 2017; Miller et al., 2013; Sieving et al., 2020). As a result, providers are missing key opportunities to provide much-needed contraceptive counseling to adolescents (Kharbanda et al., 2014). Those providers who actually do undergo specific family planning training are more likely to incorporate this counseling into their practice (Papas et al., 2017). Those patients who receive contraceptive counseling are more likely to go on to use contraception in future sexual encounters (Lee et al., 2011). The curriculum was designed assuming no prior contraception knowledge or contraceptive counseling experience, which makes it accessible to all levels of learners. The webinar component of the training is readily available to any learner and can be viewed on one’s own schedule, so can be used by any provider, nurse, or health educator in the ED wishing to add contraceptive counseling to their repertoire. This training could also be implemented among providers and staff in inpatient settings caring for female adolescents or among providers or clinicians in general (nonpediatric) ED settings. A previous study demonstrated that pediatric providers including attending physicians, fellows, and residents in the inpatient setting agree that contraception prescriptions would be appropriate in this setting (Goldstein et al., 2018). Given such, future studies should aim to replicate this study and assess its success in the inpatient setting and general ED settings, as well as at nonacademic centers.

Despite the potential for broader applicability, there are several areas of the training that can be improved. Based on limitations identified by the participants, this would include shortening the duration of the webinars, including more cultural competency-focused modules with a focus on cultural humility, and providing strategies for time management. In addition to the curriculum focusing more explicitly on cultural humility, it is important that future versions of the curriculum also account for more diverse cultural and religious views of premarital sex and contraception usage so as to make the curriculum more inclusive and broaden the target audience to include all adolescents regardless of their beliefs surrounding sex and contraception. As such, it is important for the curriculum to include explicit information on how to counsel individuals who may not believe in contraception usage and on how to respond to those who are particularly reluctant to engage in discussions regarding reproductive health. Finally, in future iterations, adding more specific content regarding side effects and contraindications to hormonal contraception usage would be helpful because these seemed to be areas of confusion for the participants.

### Limitations

There are limitations to this study both regarding the curriculum content and the evaluation methods. Regarding the curriculum content, the curriculum was designed to train providers to offer contraceptive counseling to adolescents in the ED, and as such, it may not be considered acceptable by all. Regarding our evaluation, while we included a combination of both self-report data and direct observation, we did not evaluate prior knowledge and experience using a pre-intervention survey tool, thus making it challenging to assess the direct impact of the training. Furthermore, feedback on the trainings themselves was not elicited with the post-study survey until months after the trainings had been completed, potentially making it difficult for participants to remember specific details and increasing risk of recall bias. In addition, less than half of the providers completed the post-study survey, which makes it difficult to extrapolate the results. Future evaluation efforts of the trainings should include pre-tests and post-tests regarding provider knowledge and immediate real-time feedback on the trainings in addition to the more objective observations of the actual counseling sessions.

## Conclusion

In summary, our curriculum, including both webinar content and in-person sessions, offers a novel and efficient approach to train NPs and PAs to deliver patient-centered contraception counseling to adolescents in the busy pediatric ED setting. After our training, participants successfully incorporated content and communication principles into their counseling sessions and reported feelings of improved competence and knowledge regarding contraceptive counseling. In addition, this training could be implemented in other settings and with other provider groups. Overall, there is a great need for contraception education and management, and this curriculum offers one unique approach for how to train providers to incorporate this education into the general adolescent ED visit.

## Figures and Tables

**Table 1.** Educational Objectives of the Training Curriculum

Objective Number	Learning Objective
Objective 1	Be able to discuss risks, benefits, and side effect profiles of contraceptive options with adolescents using a tiered-effectiveness approach
Objective 2	Succinctly counsel adolescents in the emergency department regarding contraceptive options
Objective 3	Ask open-ended questions and demonstrate the principles of teach-back to provide developmentally appropriate communication to adolescents
Objective 4	Use patient-centered techniques, cultural competency, and shared decision-making principles to create a safe environment in which to guide adolescents in making contraceptive choices

**Table 2.** Four Webinar Modules and Learning Objectives

Webinar Module	Webinar Learning Objectives	Running Time
Webinar 1: Understanding teen pregnancy Available at: https://www.youtube.com/watch?v=kkWYjpyXa0&list=PLuhd2MjW7f31Jcq0rgiPsjugzDEbx0BZ1&index=1	• Discuss the magnitude and repercussions of teen pregnancy in the United States • Review why the emergency department is an ideal setting for providing contraceptive counseling	11:07
Webinar 2: The psychology of contraceptive choices Available at: https://www.youtube.com/watch?v=kRNv2kQ_ZHw&list=PLuhd2MjW7f31Jcq0rgiPsjugzDEbx0BZ1&index=2	• Familiarize participants with a theoretical framework that can be used to help understand the contraceptive decision-making process for an individual • Discuss some of the barriers to and facilitators of contraceptive decision making for teenagers	10:23
Webinar 3: Exploring contraceptive options Available at: https://www.youtube.com/watch?v=KRSHSDNGI_U&list=PLuhd2MjW7f31Jcq0rgiPsjugzDEbx0BZ1&index=3	• Familiarize participants with the wide array of different contraceptive options available to teenagers • Provide participants with an efficacy-based schema for presenting information on different contraceptive options to teens • Enable participants to discuss each of the different contraceptive options in detail with their patients, including their mechanisms of action, efficacy, side effect profile, advantages, and contraindications to usage	22:45
Webinar 4: Helping teenagers navigate the contraceptive choice Available at: https://www.youtube.com/watch?v=Q8xPgfThzsg&list=PLuhd2MjW7f31Jcq0rgiPsjugzDEbx0BZ1&index=4	• Familiarize participants with the basic principles of and approach to client-centered contraceptive counseling • Provide participants with a general overarching structure for individual counseling sessions • Provide participants with tools and tricks to help them become better contractive counselors	32:20

**Table 3.** APP In-Person Training Role-Playing Scenarios

Case Number	APP Counselor	Mock Patient	Observer
1	A 17-year-old, sexually active, young woman is being seen in the ED for evaluation of abdominal pain	She knows she would like to start contraception because she is sexually active and thinks she would like birth control pills or the implant; as the counseling progresses, she is ultimately interested in an IUD. However, she has a history of migraines and is very concerned about potential side effects and risks related to her headaches	Helpful info for counseling: It is important to clarify whether her headaches are migraines with aura (visual or auditory) or any focal neurological symptoms. If her headaches don’t have those components, then the full array of options are available and the NP could focus in on what her preferences are regarding the IUD and side effects of copper vs hormonal. Use CDC MEC if needed
2	An 18-year-old young woman is being seen in the ED for a displaced forearm fracture. She reports that she is considering becoming sexually active in the near future but has not been in the past	Initially during the counseling session, she is not interested in any contraception—they will “just use condoms.” She is interested in having several children in the future and is concerned about effects on her fertility from hormonal contraception. She is also worried about “chemicals” in her body—her friends have told her that hormonal contraception is unnatural. As the counseling progresses, however, she starts considering depo	Helpful info for counseling: “When I talk with teens in the ED, many say they want to have a big family someday/have a baby while they are still young. How do you picture your future?” Try to understand her timeline on children—depo has the longest return to fertility of all the contraceptive options. The copper IUD is perhaps an ideal choice if she has a strong preference for a nonhormonal option. With more information about her preferences on those topics it would be easier to provide more tailored options. Also, the APP would of course want to encourage her to still use condoms for STI protection
3	A 16-year-old, recently sexually active, young woman is being seen in the ED for vaginal discharge. She reports that she does not intend to be sexually active again in the foreseeable future	She is very reluctant to learn about contraception at all and never reaches a point during the counseling where she will consider a particular option	Helpful info for counseling: Some teens won’t be interested no matter what we say! Consider offering up resources for her to take home (bedsider chart comparing contraceptive options, a list of adolescent/planned parenthood clinic locations, and how to obtain free condoms) in case she changes her mind

**Table 4.** Educational Objectives and Outcome Variables Used to Evaluate Training Success

Educational Objective/Goal (By the End of the Training Sessions Learners Will be Able to …)	Specific Outcome	Outcome Variable/s^a^
*Discuss the risks, benefits, and side effect* profiles of contraceptive options with adolescents using a *tiered-effectiveness approach*	Discussion of risks, benefits and side effects	Percentage of counseling sessions including risk and side effect discussion
	Usage of tiered-effectiveness counseling approach	Percentage of counseling sessions using tiered-effectiveness approach
*Succinctly* counsel adolescents in the emergency department regarding contraceptive options	Succinctness of session	Mean duration of counseling sessions
		Percentage of APPs who agree/ disagree there was adequate time for counseling session
Ask *open-ended questions* and demonstrate the principles of *teach-back* to provide developmentally appropriate communication to adolescents	Teach-back method utilization	Percentage of counseling sessions using teach back methods
	Open-ended question utilization	Percentage of counseling sessions in which open-ended questions were asked throughout the visit
Use *patient-centered* techniques, *cultural competency*, and *shared decision-making* principles to create a safe environment in which to guide adolescents in making contraceptive choices	Patient centeredness	Percentage of counseling sessions including discussion of adolescent personal goals
	Demonstration of cultural competency	Percentage of counseling sessions acknowledging cultural background in counseling
	Shared decision-making utilization	Percentage of counseling sessions involving shared decision making
	Creation of safe environment	Percentage of counseling sessions in which a safe environment was created
	Personal goals discussion	Percentage of counseling sessions in which personal goals of adolescents were discussed

a Outcome variables were obtained from the Provider Fidelity Surveys and APP Feasibility Surveys.
